# Long‐term ungrounded cable support for short‐to‐shield syndrome

**DOI:** 10.1002/ccr3.2583

**Published:** 2020-02-07

**Authors:** Brian Ayers, Christina Cheyne, Katherine Wood, Amy Quinlan, Sara Dick, Himabindu Vidula, Jeffrey Alexis, Bryan Barrus, Sunil Prasad, Igor Gosev

**Affiliations:** ^1^ University of Rochester Medical Center Rochester NY USA

**Keywords:** cardiothoracic surgery, cardiovascular disorders, critical care medicine, left ventricular assist device, mechanical circulatory support, short‐to‐shield

## Abstract

Short‐to‐shield (STS) is a potential complication for left ventricular assist device (LVAD) patients supported by the HeartMate II (HMII) pump. This phenomenon occurs when a damaged internal wire within the driveline makes contact with the surrounding sheath, resulting in insufficient power delivery to the motor when connected to a grounded power base unit (PBU). An ungrounded cable can be used to negate these effects, but the long‐term safety of this treatment strategy is unknown. In this case series, we present our institutional experience treating 17 STS patients with an ungrounded cable. In total, we present 4922 patient‐days (13.4 patient‐years) of ungrounded cable support after primary STS treatment. There were no deaths or complications related to STS. These data suggest that the long‐term use of an ungrounded cable is a reasonable treatment option for patients who cannot or do not wish to undergo pump exchange or splice repair.

## Graphical abstract

Patients that experience short‐to‐shield can be safely managed with an ungrounded cable long‐term with careful monitoring.

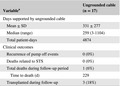

## 1 INTRODUCTION

Short‐to‐shield (STS) is an uncommon but serious complication associated with the HeartMate II (HMII, Thoratec Corp.) pump.^1^ This previously described complication occurs when one of the six internal wires in the driveline is damaged and makes contact with the surrounding silver‐coated copper braided shield.^2^ The resulting short circuit “leaks” power whenever connected to a grounded power base unit (PBU) causing insufficient power delivery to the motor and immediate cessation of pump function. The newer generation HeartMate 3 (HM3) and HeartWare HVAD (HeartWare Inc) have fixed this driveline problem and are currently the most commonly implanted pumps after demonstrating significantly better patient outcomes.^3^, ^4^ However, many patients remain on support with the HMII left ventricular assist device (LVAD) worldwide and all are at risk of developing STS, making its proper management clinically very important.^5^

There are three main treatment options for STS: (a) external splicing of the driveline lead, (b) reoperation for device exchange, or (c) use of an ungrounded cable when using the PBU. Little data exist on the optimal treatment strategy. The use of an ungrounded cable is the least invasive method but does not directly address the underlying wire fracture, making its long‐term safety unknown. In this case series, we aim to describe the long‐term outcomes of our institutional experience managing a large cohort of STS patients with an ungrounded cable.

## 2 CASE SERIES

### 2.1 Patient selection

We retrospectively reviewed a prospectively maintained database of all patients implanted with an LVAD at our institution from January 2008 through September 2018. Of the 294 patients supported by a HMII device, 30 (10%) developed STS.

Patients were grouped based on their primary treatment strategy: 9 (30%) patients underwent pump exchange, 4 (13%) patients were treated via external splicing of the driveline lead, and 17 (57%) patients were managed with an ungrounded cable. All patients treated with an ungrounded cable were included in this study.

### 2.2 Patient characteristics

**Figure 1:**
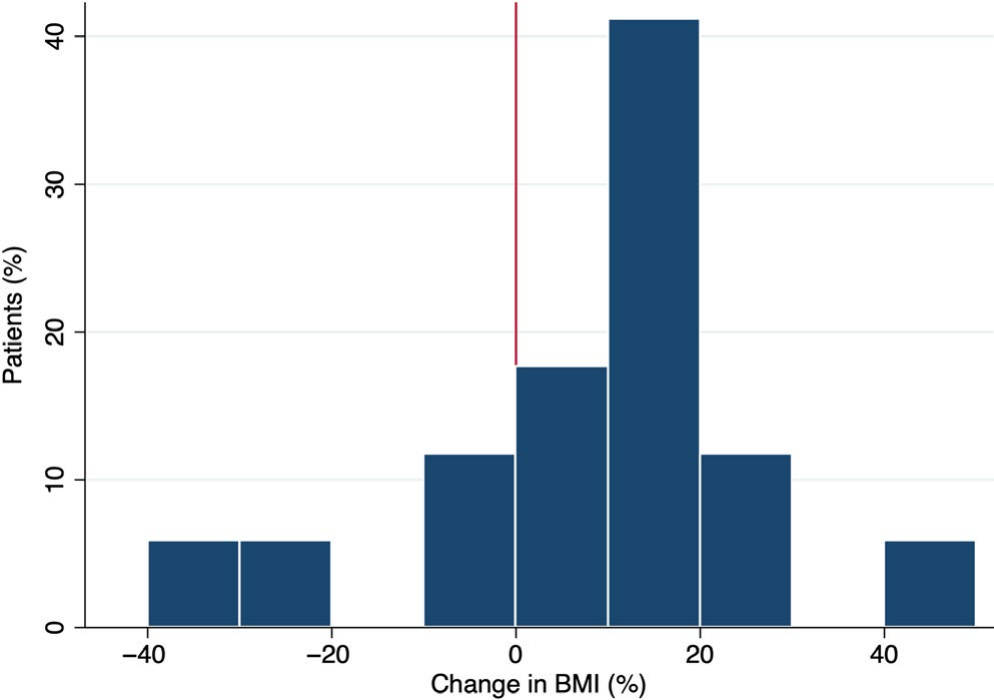
Change in body mass index (BMI) from time of LVAD implantation to development of short‐to‐shield syndrome

Baseline characteristics of patients at time of LVAD implantation are summarized in Table 1. Patients were predominately male (76%) with nonischemic cardiomyopathy (65%) and median age of 54 years (range, 26‐69 years). Body mass index (BMI) at time of implant ranged from 21 to 43 kg/m^2^ (mean ± SD, 31 ± 5.7 kg/m^2^). Mean time from implant to STS diagnosis was 1009 ± 651 days (range, 249‐2484 days). A majority of the patients (76%) experienced weight gain from time of LVAD implantation to developing STS (Figure 1).

**Table 1:** Patient characteristics

Variable^a^	Ungrounded cable (n = 17)
Male	13 (76%)
Age (y)	52.3 ± 12.7, (26‐69)
Preimplant BMI (kg/m^2^)	31.0 ± 5.7, (21‐43)
Heart failure etiology	
Ischemic	6 (35%)
Nonischemic	11 (65%)
Diabetes	4 (24%)
Prior cardiac surgery	4 (24%)
Delayed closure at time of implant	6 (35%)
INTERMACS at implant	
Profile 1	3 (18%)
Profile 2	3 (18%)
Profile 3+	11 (64%)
Therapy strategy	
Bridge to transplant	5 (29%)
Destination therapy	12 (71%)
Time from implant to STS (d)	1009 ± 651, (249‐2484)

Abbreviations: BMI, body mass index; STS, short‐to‐shield.

a Values presented as no. (%) or Mean ± SD, (range).

### 2.3 Identification and management of STS

When a patient presents with potential STS symptoms (most commonly position‐dependent pump off events or low‐speed advisories), the logfile is examined and radiographic imaging is sent to the Abbott engineering team to look for potential visible points of shield breakdown. If the damage is suspected to be extracorporeal, then splicing of the driveline is typically performed. If external splice repair is felt to not be a viable option, then a shared‐decision‐making discussion is held with the patient to discuss the risks and benefits of pump exchange compared with ungrounded cable. In this series, 17 (57%) of the patients presenting with STS elected to undergo long‐term support with an ungrounded cable.

### 2.4 Complications and outcomes

Total available follow‐up data represent 4874 patient‐days (13.4 patient‐years) of ungrounded cable support (Table 2). Average follow‐up time supported by an ungrounded cable was 331 ± 277 days per patient. One patient (6%) died of unrelated causes, and three (18%) patients underwent orthotopic heart transplantation. There were no STS‐related complications or deaths during the follow‐up period.

**Table 2:** Long‐term follow‐up for patients supported by an ungrounded cable

Variable^a^	Ungrounded cable (n = 17)
Days supported by ungrounded cable	
Mean ± SD	331 ± 277
Median (range)	259 (3‐1104)
Total patient‐days	4874
Clinical outcomes	
Recurrence of pump off events	0 (0%)
Deaths related to STS	0 (0%)
Total deaths during follow‐up period	1 (6%)
Time to death (d)	229
Transplanted during follow‐up	3 (18%)

a Variables presented as no. (%) or otherwise indicated.

## 3 DISCUSSION

Short‐to‐shield is an uncommon but serious complication associated with the HeartMate II pump. It occurs in up to 9% of patients supported by a HMII device.^6^ Our data support previous reports that have shown a majority of patients that develop STS have experienced weight gain since time of LVAD implantation.^7^ A theory is that large changes in body composition can alter the optimal angle for the driveline, resulting in points of increased tension and subsequent driveline fracture. Increased physical function status has also been suggested to be associated with STS, as more active patients may place increased mechanical stresses on the driveline.^7^ More research is needed to determine other potential patient characteristics associated with an increased risk of developing STS.

The three main treatment options for STS consist of (a) external splicing of the driveline lead, (b) pump exchange, or (c) use of an ungrounded cable. Currently, there is a paucity of data in the literature regarding the optimal treatment for patients experiencing STS. From our institutional experience, we have developed a treatment model for STS that relies on a shared‐decision‐making approach. If a patient's driveline damage is identified in an extracorporeal location, then splicing of the driveline is typically performed. Splice repair of the driveline is a noninvasive procedure that has the potential to fix the underlying damage associated with STS. Stulak et al presented data suggesting it is an effective and durable long‐term solution associated with a low incidence of complications (20% required reintervention).^8^ Similarly, Pal et al found that 10% of 321 splice repairs had a serious malfunction.^9^ Coyle et al, however, present a different institutional experience that found splice repair to be an ineffective and short‐term solution with 89% of repairs requiring an additional subsequent intervention.^7^ Moreover, it is important to note that the splice repair procedure itself is also associated with substantial, including complete pump failure and abrupt pump stoppage from lack of power delivery.^9^ Our institutional policy is to always have extracorporeal membrane oxygenation (ECMO) on standby and an operating room available for emergent exchange if needed when performing any splice repair. It is important to properly consent the patient to the risks of the procedure and be prepared to move emergently to pump exchange whether there is a major complication.

If external splice repair is felt to not be a viable option, then a shared‐decision‐making discussion is held with the patient to discuss the risks and benefits of pump exchange compared with ungrounded cable. Multiple studies have demonstrated that device exchange can be done with relatively low rate of perioperative complications or mortality.^10^, ^11^, ^12^, ^13^ Pump exchange has the benefit of definitively fixing the damage, but does carry the inherent risks associated with undergoing an operation and may be associated with an increased risk of postoperative infection or recurrent thrombosis.^14^ However, another potential benefit is that patients undergoing pump exchange can be upgraded from a HMII to a newer generation pump (HM3 or HVAD), thus decreasing their risk of device malfunction and pump thrombosis. Early reports suggest this procedure is safe and can be accomplished through less invasive approaches that do not require a reoperative full sternotomy.^15^

For patients that are high‐risk surgical candidates or do not wish to undergo another operation due to personal preference, the use of an ungrounded cable is a viable option. The supplied grounded PBU system is replaced with an ungrounded PBU to avoid the short‐circuit effect caused by the damaged internal wire. The benefit is that the patient can continue LVAD support without an operation, but it does not fix the underlying wire damage. The predominate concern is if a second internal wire is damaged then a phase‐to‐phase electrical short may develop and cause the pump to lose all power. Clinicians may be hesitant to use ungrounded cables long‐term due to the present uncertainty regarding the likelihood of damage to a second internal lead. To date, there is only one case series in the literature describing a single center's experience with long‐term ungrounded cable support, which demonstrated 28% of patients developed a subsequent phase‐to‐phase short while on an ungrounded cable.^7^ However, all cases were managed successfully with subsequent intervention and did not result in any patient deaths. Our data support these findings, suggesting that ungrounded cable support has a low incidence of subsequent complications and is a reasonable, noninvasive management strategy for STS long‐term.

## 4 LIMITATIONS

The main limitation of this study is that it is retrospective and observational in nature. While this is the largest reported STS cohort supported by an ungrounded cable to date, it is still a relatively small number of patients. The lack of complications or mortality seen in this study should be interpreted with caution and does not imply that the use of an ungrounded cable is without risks. Each treatment option for STS is associated with its own unique set of risks and benefits. We advocate for a shared‐decision model with the patient in order to select the management plan that best aligns with the individual's personal goals.

## 5 CONCLUSIONS

In this study, we present a large cohort of STS patients treated with an ungrounded cable with over 13.4 patient‐years of follow‐up. There have been no complications or patient deaths related to STS while supported by the ungrounded cable. These data suggest the long‐term use of an ungrounded cable a reasonable treatment option for those patients who cannot or do not wish to undergo pump exchange or splice repair. Management of short‐to‐shield should involve a patient‐centered approach to determine which management strategy best aligns with their goals and priorities.

## CONFLICT OF INTEREST

Amy Quinlan is a consultant for Abbott. Igor Gosv is a consultant for Abbott. Sunil Prasad is on the scientific advisory board for Abbott. No other authors have disclosures.

## AUTHOR CONTRIBUTIONS

BA, CC, and IG: substantially contributed to conception and design of study, acquisition, analysis, and interpretation of data, and involved in drafting and revising the manuscript. KW, AQ, SD, HV, JA, BB and SP: substantially contributed to conception and design of study, and involved in drafting and revising the manuscript.
